# Towards Eliminating Bias in Cluster Analysis of TB Genotyped Data

**DOI:** 10.1371/journal.pone.0034109

**Published:** 2012-03-29

**Authors:** Cari van Schalkwyk, Madeleine Cule, Alex Welte, Paul van Helden, Gian van der Spuy, Pieter Uys

**Affiliations:** 1 The South African Department of Science and Technology/National Research Foundation (DST/NRF) Centre of Excellence in Epidemiological Modelling and Analysis, Faculty of Science, University of Stellenbosch, Stellenbosch, South Africa; 2 African Institute for Mathematical Sciences, Muizenberg, South Africa; 3 Department of Statistics, University of Oxford, Oxford, United Kingdom; 4 DST/NRF Centre of Excellence in Biomedical Tuberculosis Research/MRC Centre of Molecular and Cellular Biology, Division of Molecular Biology and Human Genetics, Faculty of Health Sciences, University of Stellenbosch, Stellenbosch, South Africa; McGill University, Canada

## Abstract

The relative contributions of transmission and reactivation of latent infection to TB cases observed clinically has been reported in many situations, but always with some uncertainty. Genotyped data from TB organisms obtained from patients have been used as the basis for heuristic distinctions between circulating (clustered strains) and reactivated infections (unclustered strains). Naïve methods previously applied to the analysis of such data are known to provide biased estimates of the proportion of unclustered cases. The hypergeometric distribution, which generates probabilities of observing clusters of a given size as realized clusters of all possible sizes, is analyzed in this paper to yield a formal estimator for genotype cluster sizes. Subtle aspects of numerical stability, bias, and variance are explored. This formal estimator is seen to be stable with respect to the epidemiologically interesting properties of the cluster size distribution (the number of clusters and the number of singletons) though it does not yield satisfactory estimates of the number of clusters of larger sizes. The problem that even complete coverage of genotyping, in a practical sampling frame, will only provide a partial view of the actual transmission network remains to be explored.

## Introduction

In order to better understand the epidemiology of tuberculosis (TB), recent infection needs to be distinguished from the reactivation of latent disease, for instance to assess the success of intervention programs. To this end, molecular techniques of DNA ‘fingerprinting’ such as restriction fragment length polymorphism (RFLP) are commonly used. Typically, bacteria from a sample of infected individuals are typed, and classified as either ‘clustered’ or ‘unique’. Unique cases each form what is termed a ‘singleton cluster’. Two cases yielding the same type, and hence in the same cluster, are usually considered likely to be directly ‘linked’ in the following sense: either one case is the ‘descendant’ of the other, or they share a common ‘ancestor’ [1], [2]. Hence, the proportion of clustered individuals is used as an indicator of the proportion of on-going or recent transmission.

There are two common rules of thumb for estimating the proportion of cases due to recent transmission: the ‘n method’ and the ‘n-1 method’. The former uses the proportion of cases in clusters as a proxy for the proportion of cases due to recent transmission. In the latter, one case from each cluster is assumed to be an index case, and the proportion of non-index cases is used as a measure of recent transmission (thus the ‘n-1 method’ always leads to a lower estimate of the proportion of recent transmission).

It is unlikely that one will be able to identify every active case in a community. Moreover, among sputum confirmed TB subjects encountered (typically self-reporting to clinics), not all sputum samples will be successfully typed. However, the proportion successfully typed (sampling rate) is, of course, known.

It has previously been shown [3], [4] that naïve estimates of clustering exhibit a systematic bias, leading to underestimation of the proportion of clustered individuals. There are three components to this problem of bias: 1) the imperfect view of the epidemic in a community provided by considering only the reported TB cases for a given finite period of time e.g. bias caused by under-diagnosis, partial contact tracing or the restriction of the time window. For these and other various logistical reasons, the reported cases do not represent a random sample from all TB cases in the community. 2) The sample of genotyped cases is not necessarily a random sample of the reported cases due to the diagnostic probability of culture-positivity being dependent on age and HIV status. To reduce this bias, children should be excluded from the study population. In settings where HIV prevalence is high, this bias will not be negligible. 3) Bias in the number of unique cases exists due to contributions resulting from sampling the larger clusters, e.g. 10 clusters of size 4 when sampled at a rate of 0.6 may present as 4 singleton clusters (uniques) together with 3 doublet clusters, 1 triplet cluster and 2 clusters of size 4. For the same reason, bias arises in the total number of clusters. These contributions to total bias are not insignificant. This third source of bias will be called frequency distribution bias.

An estimation method to eliminate the bias in 3) only is demonstrated. This method makes no attempt to address the bias in 1) above, nor the bias in 2). Should, however, the notified cases form a random sample of TB cases in the community, and the genotyped cases a random sample of notified cases, then the present analysis could be used to make inferences about transmission in the community. In the remainder of this paper, it is assumed that genotyped cases do form a random sample of the notified cases so that the method addresses the question of the proportion of transmission represented among the notified cases only.

Four existing datasets are used to illustrate this method. In addition to a less biased estimate for the amount of clustering, an estimate of the variance, and hence confidence intervals, is obtained. However it must be noted that these results effectively assume no bias of type 2.

In subsequent sections the term ‘population’ will refer to all individuals in a community for whom a sputum-based positive TB diagnosis was made. The group for whom sputum samples were successfully typed will be called the ‘sample’. Bias refers to the frequency distribution bias.

## Methods

### Notation and preliminaries

Note the following definitions that hold for the population:

- Each case in the population is a member of a cluster.
- Let *M* be the number of clusters, with the typical cluster indexed by *i = 1*,…, *M* , and let *a_i_* be the size of the *i* ^th^ cluster.
- *a*  =  (*a*_1_, … , *a_M_*) therefore represents the sizes of the clusters in the population.
- The total number of cases is given by 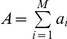.
- Let *A_k_* be the total number of clusters of size *k*, (*k*  =  1, …, *N*  =  max(*a_i_*)).
- The population vector of cluster size frequencies is then given by ***A***  =  (*A*_1_, … , *A_N_*).

*S* of the *A* total cases are typed and clustered using genotyping. It is assumed that the sampling process is independent of the clusters i.e. each case is equally likely to be typed, irrespective of which cluster it belongs to. This gives rise to observed clusters of size ***s***  =  (*s*_1_, …, *s_M_*). The investigator is of course unaware of the existence of clusters which have an observed size of zero. Nevertheless, ***s*** can be thus defined and has a multivariate hypergeometric distribution, with mass function
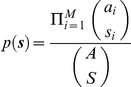



The value
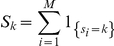
represents the total number of clusters of size *k* observed in the typed sample. 1 denotes the indicator function, that is,
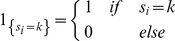
The sample vector of cluster frequencies (a histogram of observed cluster sizes) is given by: 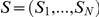. Of course, it is not possible to know *N*, the true size of the largest cluster. Thus, a truncated vector 
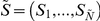
 is observed, where 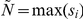 is the largest observed cluster size.

Let **P** denote the matrix
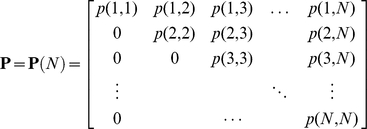
where
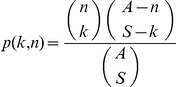
is the hypergeometric probability mass function, which represents the probability that a population cluster of size *n* presents as a cluster of size *k* in the sample.

It is additionally useful to define the probability that population clusters of size *m* and *n* present as sample clusters of size *j* and *k* respectively:
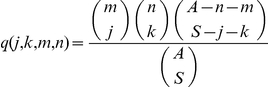



### Quantities of interest

The main quantities of interest are:

1. *M* , the total number of clusters
2. *A*_1_, the number of unclustered cases (i.e. the number of singleton clusters)

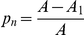
, the proportion of cases not in singleton clusters. According to the ‘n method’ heuristic, this is the proportion of recent transmissions.

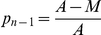
, the proportion of cases which are not the first case in a cluster. According to the ‘n-1 method’ heuristic, this is the proportion of recent transmissions.

Naïvely, one would estimate 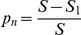 and 
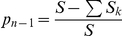
. These estimators have been shown to be biased [3], [4]. In the subsequent section, unbiased estimators are derived for *M* and *A*_1_, whence estimators for *p_n_* and *p_n-1_* may be directly derived by substitution into their definitions (*A* is simply the reported number of positive TB diagnoses in the study). The uncertainty (standard error) inherent in the estimator is also analyzed in order to obtain confidence intervals.

### Estimator of *M* and *A*_1_

It can be shown that (see Supporting Information S1), for *k*  =  1, … , *N*,
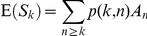
or, in matrix notation,
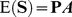
(1)It can also be shown that the diagonal elements of the covariance matrix, cov(*S*)*_kk_* are given by:
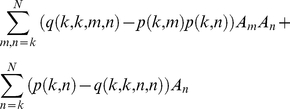
(2)And the off-diagonal elements cov(*S*)*_kl_* by:
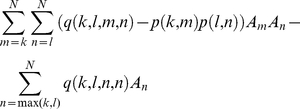
(3)From equation 1, a crude estimate 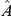 of ***A*** may be obtained by simply matching the first moment, that is,
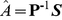
(4)The covariance matrix of 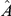 may be calculated using equations 2 and 3. Due to high variance and high correlation between components, this performs poorly as an estimate of ***A***. The origin and consequences of this inherent instability are discussed in the Supporting Information S2.

The crucial quantities of interest are the total number of clusters 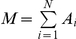
and the number of singletons, *A*_1_. An estimator of *M* can be derived as:
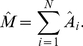
(5)The first element of the vector 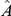 is an estimate for *A*_1_. These are unbiased as a consequence of equation 1. Expressions for the variances 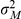 and 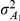 may be derived from equations 2 and 3. These quantities still depend on the (unknown) ***A***. The estimate of 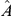 =  **P^−1^** ***S*** can be used to obtain estimates 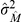 and 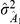 of 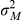 and 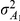.

### Approximate confidence intervals

Since 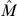 is a linear combination of random variables (albeit with some dependence), a normal approximation to the distribution of 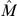 seems reasonable. Assuming this approximation is valid, the estimate 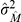 of 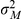 can be used, which leads to the approximate (1 - α) - level confidence interval of
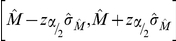
Similarly, an approximate (1 - α) - level confidence interval for *A*_1_ is given by:
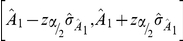



### Computational considerations

Note that the estimates in equations 4 and 5 still involve an unknown quantity, namely *N*, the maximum population cluster size. However, from the upper triangular structure of **P** and the fact that *S_n_*  =  0 for 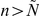, the estimates are unchanged if **P**(*N*) is replaced by **P**(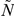) and ***S*** by the observed vector 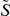.

The matrix **P** is close to singular when 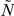 is large. A truncation approach to the use of the **P** matrix is now introduced. The observed vector 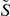 is divided into two parts ***S***_0_ (the first *C* components, which lead to a numerically stable inversion of **P**) and ***S***_1_ (the remaining 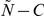
components). The vector ***S***_0_ is used as the input into the method outlined above, and the number of clusters in ***S***_1_ is simply a known number of clusters to be added to any inferred total cluster count. The key question that arises is whether the truncation leads to stable estimates of the number of clusters, *M*, and the number of singletons, *A*_1_, and this is investigated below.

The maximum cluster size for which the **P** matrix is still numerically non-singular will be the maximum cluster size at which the vector ***S***_0_ can be truncated. Within this range, it is now possible to explore bias and variance of estimates of *M* and *A*_1_. This is done for hypothetical populations at various sampling rates and truncations under Results.

## Results

### Exploring bias and variance of *M* and *A_1_*

**Figure 1:**
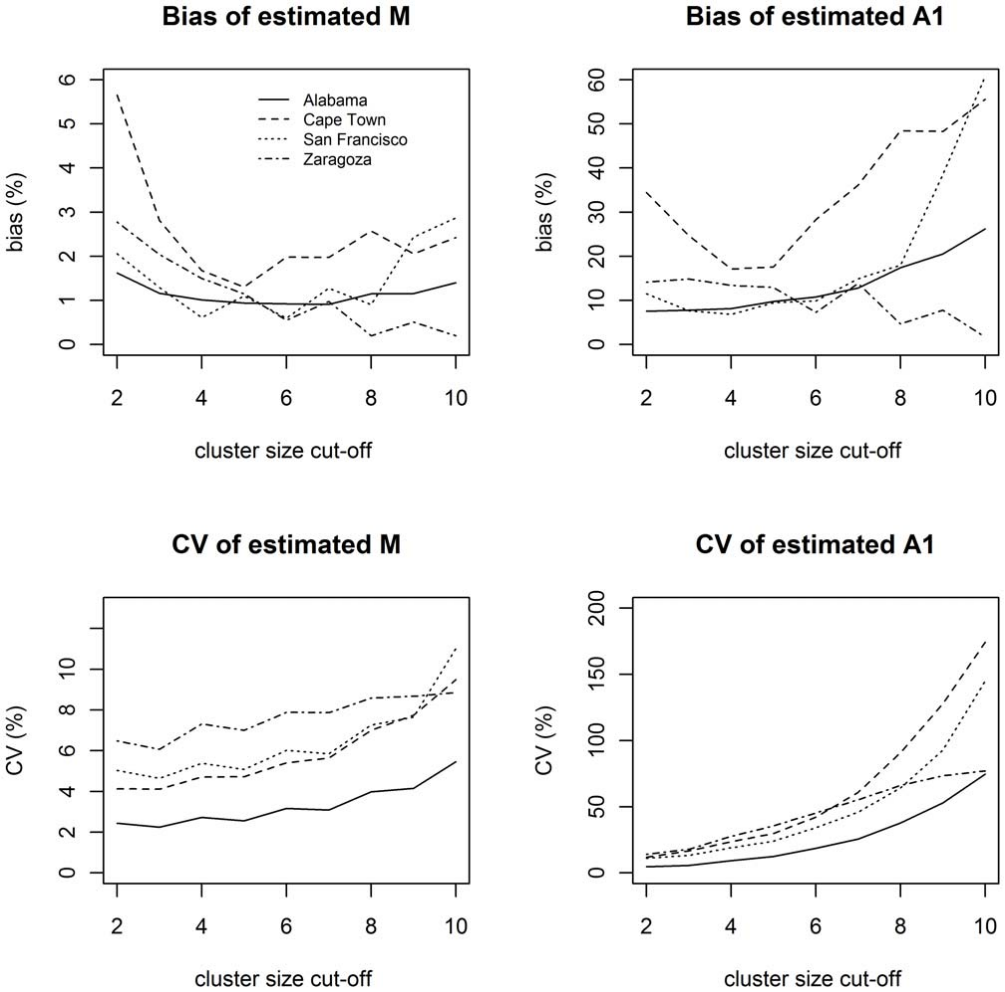
Normalized bias and coefficient of variation for estimated M and A_1_ for sampling rate of 40%. The normalized bias alternates between positive and negative values at even and uneven truncations respectively. For ease of viewing, the absolute values of normalized bias are shown.

**Figure 2:**
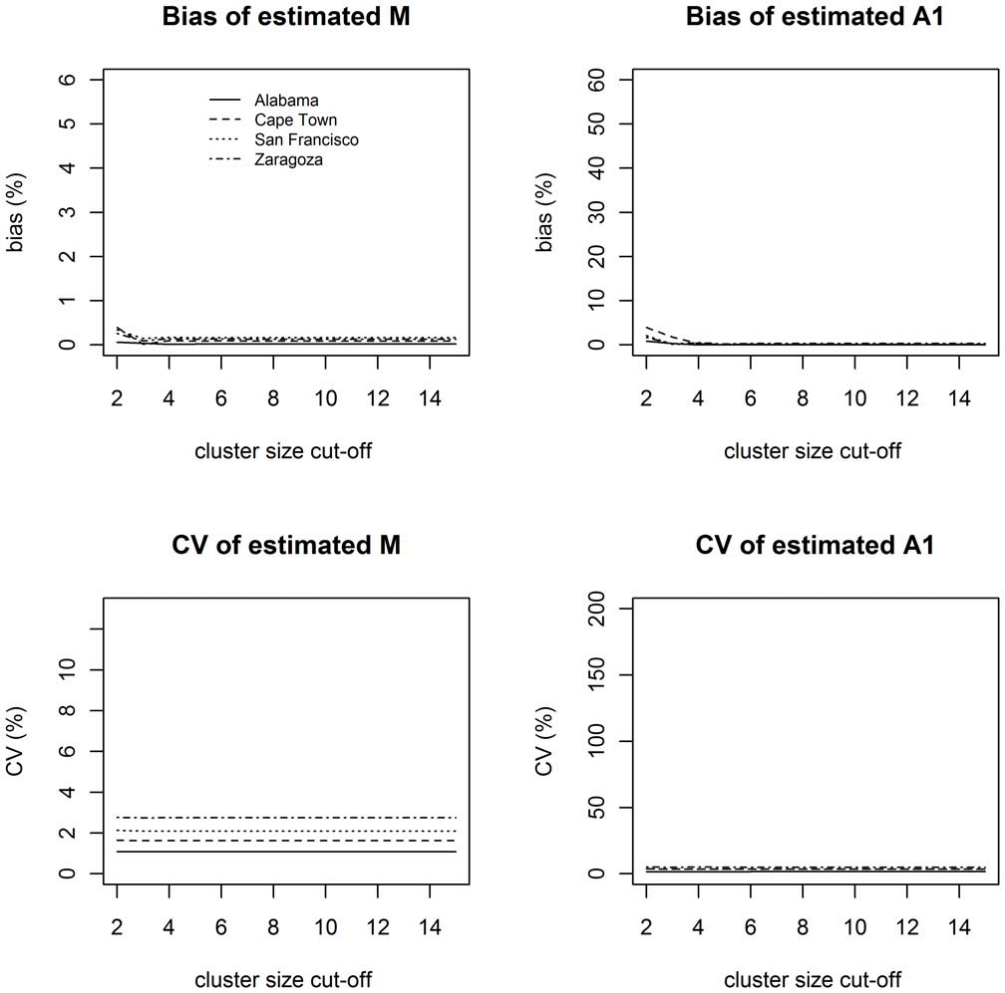
Normalized bias and coefficient of variation for estimated M and A_1_ for sampling rate of 70%. The maximum normalized bias at truncation cluster size of 6 is 0.164% for M and 0.299% for A_1_. The maximum CV at the same truncation is 2.75% for M and 4.92% for A_1_. The normalized bias alternates between positive and negative values at even and uneven truncations respectively. For ease of viewing, the absolute values of normalized bias are shown.

In order to explore this bias and variance, suitable hypothetical populations are required since actual populations are not known. Available data from 4 cities (Alabama [5], Cape Town [6], San Francisco [1], Zaragoza [7]) were used to generate hypothetical populations which produce samples clustered close to the observed data sets (see the method and data in Supporting Information S3). One thousand samples for each of these populations were obtained by sampling according to the multivariate hypergeometric distribution given in Equation 1. (The statistical software R version 2.14.1 was used for all analyses.) The **P** matrix inversion method described above was used to obtain estimates for the number of clusters, *M* , and the number of singletons, *A*_1_. The coefficient of variation (CV) and the absolute of normalized bias for sampling rates of 40% and 70% are illustrated in Figure 1 and Figure 2, respectively.

At low sampling rates the estimates of the number of clusters, *M*, are more stable than the estimates of the number of singletons, *A*_1_. The relative variability for both *M* and *A*_1_ increases as the truncation increases. The bias for *M* is very small, and shows initial decrease with increasing truncation. The bias for *A*_1_, which is considerably higher than the bias for *M*, increases with increasing truncation. This may be an indication that estimates for *A*_1_ are not very reliable at low sampling rates.

In Figure 2 a higher sampling rate of 70% is considered. The graphs are plotted on the same scales as in Figure 1 to illustrate that bias and variability decreases dramatically for this higher sampling rate. Interestingly, the bias and variability also show no dependence on truncation.

**Figure 3:**
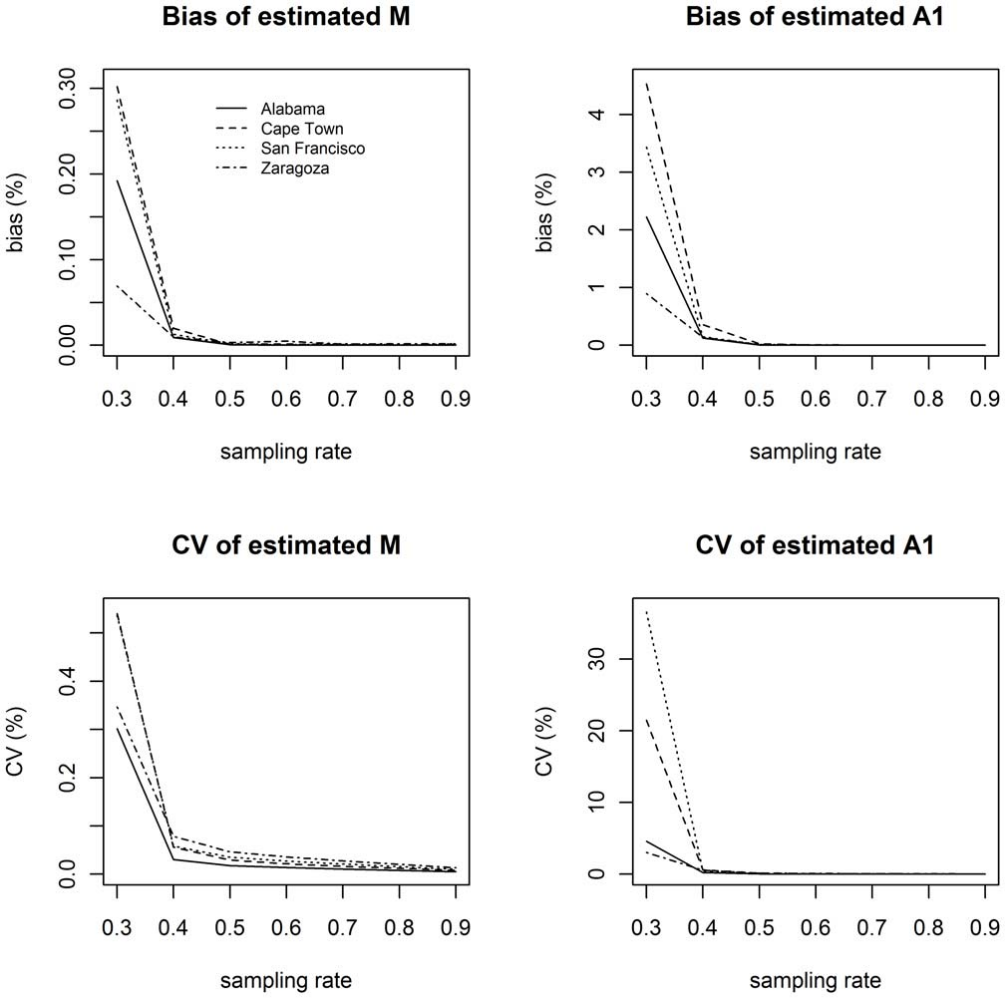
Normalized bias and coefficient of variation for estimated M and A_1_ when the vector S is truncated at size 6. The maximum normalized bias at a sampling rate of 50% is 0.206% for M and 2.38% for A_1_. The maximum CV at the same sampling rate is 4.72% for M and 13.93% for A_1_.

Figure 3 illustrates the decrease in bias and variability at a fixed truncation of 6 for various sampling rates, showing that both are very small for sampling rates of 50% and greater.

**Figure 4:**
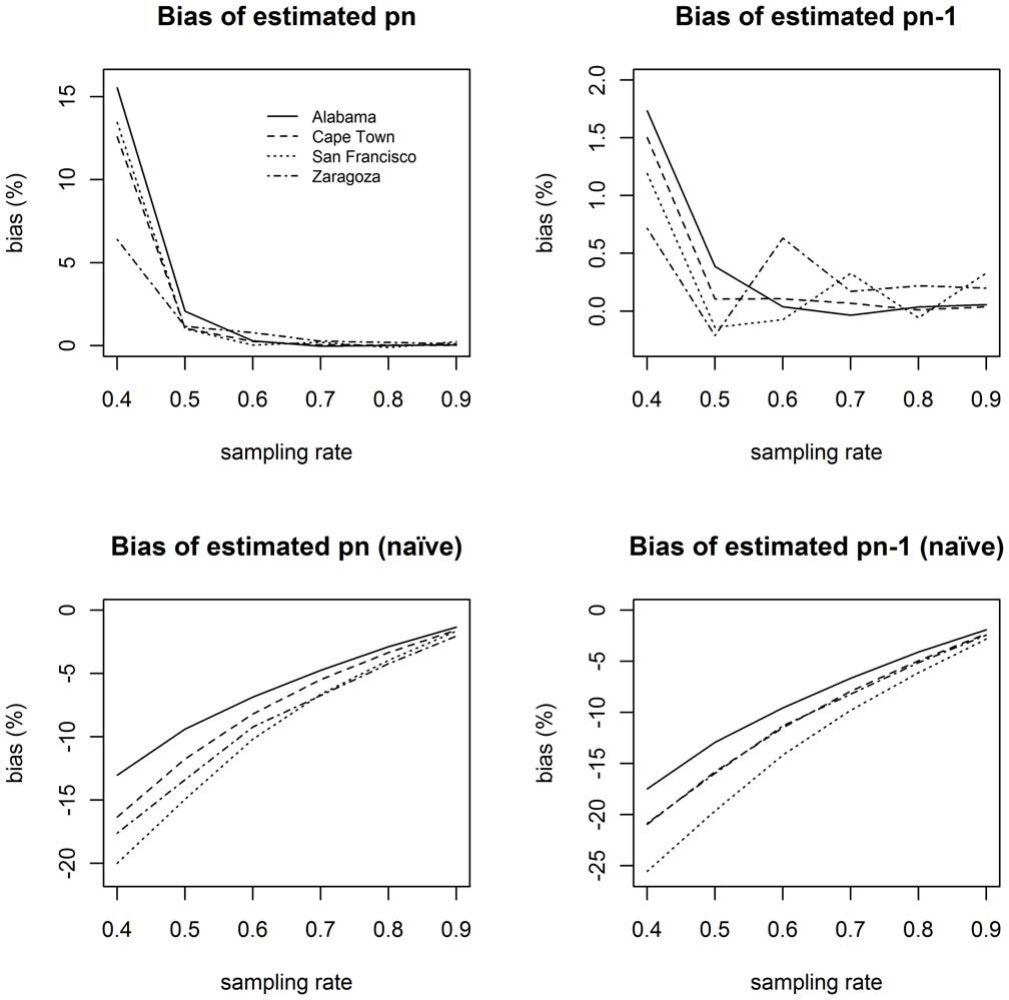
Bias in proportions using the naïve method, or the P matrix inversion method when the vector S is truncated at size 6.

Figure 4 shows that the bias in the estimate of the proportion of cases which are not singletons, 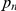, using the **P** matrix inversion method, drops to almost zero for sampling rates of 50% and higher, while the decrease in bias for the naïve method is much slower. Similarly, the bias in the estimate of 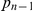 , by the **P** matrix inversion method, is less than 2% at the low sampling rate of 40% and drops to almost zero for sampling rates of 50% and higher, while the decrease in bias for the naïve method is again much slower.

The instabilities introduced by this approach could in principle be addressed by adopting a maximum likelihood or fully Bayesian approach to the inference. Given that 1) the method is quite stable in the needed regime, 2) the elements of **P** are ‘cluster level’ likelihoods and the full ‘cluster size histogram’ level likelihood analysis is therefore considerably more complicated, and 3) this additional complexity would still not address the inherent limitations of the sampling frame, there is probably no real benefit in pursuing these more general approaches.

### Applying method to existing data

The **P** matrix inversion method is applied to the same datasets considered above. In these datasets, 70% or more of TB diagnoses were typed. Based on the conclusion drawn in the previous section, any truncation can be chosen with negligible risk of introducing significant bias or variance. The results obtained effectively assume no bias of type 2). Table 1 shows the fraction of patients sampled, followed by estimates (and standard errors) for the total number of clusters, the number of clusters of size one, and the fraction, *p*, of transmitted cases, according to the ‘n method’ and ‘n-1 method’. In addition to the **P** matrix inversion method, these fractions are also calculated with the naïve method. The observed vector ***S*** is truncated at cluster size 6. The R code used to produce Table 1 is provided in the Supporting Information S3.

**Table 1:** Estimated quantities, with standard errors for truncation  =  6.

Datasets	*A*	*r*	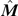	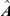	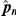	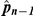	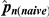	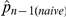
**Alabama**	2204	0.8	1408 (11.7)	1271 (14.8)	0.422 (0.0067)	0.359 (0.0053)	0.41	0.345
**Cape Town**	2093	0.7	895 (14.7)	636 (23.04)	0.696 (0.011)	0.572 (0.007)	0.653	0.528
**San Francisco**	585	0.81	391 (5.86)	339 (7.79)	0.419 (0.0133)	0.33 (0.01)	0.404	0.311
**Zaragoza**	486	0.93	276 (3.04)	227 (4.09)	0.534 (0.0083)	0.434 (0.0062)	0.526	0.427

Note: These estimates are for the notified cases only, under the assumption of no type 2 bias.

## Discussion

Cluster analysis is used to estimate the proportion of transmission of tuberculosis in a community. However, it is subject to limitations, many of which have been discussed elsewhere [3], [4], [8]. Previous attempts at assessing the magnitude of bias in the proportion of transmission failed to adequately distinguish between three distinct sources of bias: 1) the cases reporting to clinics are unlikely to represent a random sample of all active TB cases in the community, 2) the genotyped cases are not necessarily a random sample of the reported cases, 3) frequency distribution bias. The extent of bias of types 1) and 2) relative to bias of type 3) will vary according to local conditions and no general statement concerning this extent is possible here.

The present work identifies these separate problems, but solves the third problem only, under the assumption that the subset is random.

Given genotyped data on at least a majority of positive TB diagnoses (within a defined sampling frame) this work presents a method of inferring, robustly, the number of singletons and clusters that would have been observed if all positive sputa had been genotyped. This leads to an unbiased estimate of the proportion of transmission among the notified TB cases in the community.

It should be noted however, that the genotype methods currently used do not have perfect sensitivity and specificity. The choice of method necessarily results in a compromise between an evolutionary rate that is fast enough so as to provide sufficient discriminatory power between unrelated disease cases and yet still link related cases. Therefore measures of recent transmission that account for genetic heterogeneity and fingerprint pattern change rate need to be developed to ensure that the sample cluster distribution accurately represents the reality in the population [9]. Scott et al [10] investigated and compared three measures – IS*6110* RFLP, both dichotomous and continuous (nearest genetic distance) and PCR-based. They concluded that the poor sensitivity of the standard IS6110 RFLP test leads to estimates of clustering that are likely too low yet IS6110 typing remains the best method, at least in a low-incidence setting where the population of M. tuberculosis isolates shows a high degree of genetic diversity. This is in large part because IS6110 typing has the slowest evolution rate. The **P** matrix inversion method assumes that typing is accurate.

It should, moreover, also be noted that not all cases in a cluster are necessarily related by infection events. It is possible for a case to be the result of re-activation, i.e. to be endogenous, and to be misinterpreted. Thus bias lowering the number of singletons may be present. These considerations are investigated by Pretorius et al [11] and do not form part of the present work.

Knowledge of the relative impact of transmission, versus reactivation disease, can be used to design and evaluate transmission reduction programs, and target vulnerable locations or regions with appropriate interventions. This may be particularly appropriate for investigating antibiotic resistance worldwide, to explore the extent to which resistant strains are actively circulating in the community, or emerging de novo in sub-optimally treated patients. Cluster analysis is an invaluable tool to assist such investigations.

## Supporting Information

Supporting Information S1 This file describes the derivation of Equations (1)–(3).
(DOC)

Supporting Information S2 This file describes the origin and consequences of the inherent instability in estimating the vector A with equation (4).
(DOC)

Supporting Information S3 This file provides the algorithm for creating hypothetical populations for a given sample and code to reproduce the results in Table 1.
(DOC)
