# Supplementary material for: Towards Eliminating Bias in Cluster Analysis of TB Genotyped Data
Source: PLoS One. 2012 Mar 29;7(3):e34109. doi: 10.1371/journal.pone.0034109 (PMC3315507; doi:10.1371/journal.pone.0034109)
Supplement: Supporting Information S1 — This file describes the derivation of Equations (1)–(3). (DOC) [file pone.0034109.s001.doc]

# Supporting Information S1

For the mean:

E(*S_k_*)*=* $E\left(\sum_{i=1}^{M} 1_{\left\{s_{i}=k\right\}}\right)$

= $\sum_{i=1}^{M} p(k,a_{i})$

= $\sum_{n=k}^{N} p(k,n)A_{n}$

For *k* ≠ *l* ,

cov(*S_k_ , S_l_*) = E(*S_k_S_l_*) – E(*S_k_*)E(*S_l_*)

= E$\left(\sum_{i=1}^{M} \sum_{j=1}^{M} 1_{\left\{s_{i}=k,s_{k}=l\right\}}\right)-\sum_{i=1}^{M} \sum_{j=1}^{M} p(k,a_{i})p(l,a_{j})$

= $\sum_{i\neq j} q(k,l,a_{i},a_{j})-\sum_{m=k}^{N} \sum_{n=l}^{N} p(k,m)p(l,n)A_{m}A_{n}$

= $\sum_{m=k}^{N} \sum_{n=l}^{N} \left(q(k,l,m,n)-p(k,m)p(l,n)\right)A_{m}A_{n}-\sum_{n=\max(k,l)}^{N} q(k,l,n,n)A_{n}$

For *k = l*,

var(*S_k_*) = E(*S_k_*) – (E(*S_k_*))^2^

= E$\left(\sum_{i,j=1}^{M} 1_{\left\{s_{i}=k,s_{j}=k\right\}}\right)-\sum_{m,n=k}^{N} p(k,m)p(k,n)A_{m}A_{n}$

= $\sum_{i\neq j} q(k,k,a_{i},a_{j})+\sum_{i} p(k,a_{i})-\sum_{m,n=k}^{N} p(k,m)p(k,n)A_{m}A_{n}$

= $\sum_{m,n=k}^{N} \left(q\left(k,k,m,n\right)-p(k,m)p(k,n)\right)A_{m}A_{n}+\sum_{m=k}^{N} \left(p(k,m)-q(k,k,m,m)\right)A_{m}$

For variance of *p_n_*,

var(*p_n_*) = var$\left(\frac{A-A_{1}}{A}\right)$= $\frac{1}{A^{2}}$var$(A_{1})$

Similarly, variance of *p_n-1_* is: var(*p_n-1_*) = $\frac{1}{A^{2}}$var$(M)$
