# Supplementary material for: Towards Eliminating Bias in Cluster Analysis of TB Genotyped Data
Source: PLoS One. 2012 Mar 29;7(3):e34109. doi: 10.1371/journal.pone.0034109 (PMC3315507; doi:10.1371/journal.pone.0034109)
Supplement: Supporting Information S2 — This file describes the origin and consequences of the inherent instability in estimating the vector A with equation (4). (DOC) [file pone.0034109.s002.doc]

# Supporting Information S2

Relationships such as equation 4 can be used to define stable estimators when observed data are distributed in a suitably benign way around the expectation value. In the present case, there is a maximal *observed* cluster size ($\tilde{N}$), which is *extremely* unlikely to be the real maximum cluster size (*N*). The inversion implied in equation 4 is unstable with respect to the fluctuations from the small expected values (the last *N*-$\tilde{N}$entries of the vector E(***S***)) to the zeroes observed as frequencies of clusters of size greater than $\tilde{N}$ in the vector ***S***. This can be understood as the implicit truncation of the cluster size range in the ‘estimated’ distribution, which occurs at the largest observed cluster size. It is not difficult to see how the estimates of ***A*** are destabilized:

- – where **P**$(\tilde{N},\tilde{N}$becomes ever smaller for larger values of the observed maximum cluster size, $\tilde{N}$.
- Hence, by disallowing the interpretation of clusters observed at size $\tilde{N}$, as manifestations of clusters actually greater in size than $\tilde{N}$, a spuriously (very) large estimate of $A_{\tilde{N}}$ is produced.
- This in turn typically leads to unphysical (negative) estimates of other components of $\hat{A}$.

The following example illustrates these instabilities. Suppose the population vector ***A*** = [260, 95, 46, 15, 8, 3, 1, 0, 1, 1] i.e. there are 260 singletons in the population and one cluster of size 10, the maximum cluster size. If the sampling rate from this population is 60% and equation 1 is applied, the vector E(***S***) can be calculated as [217.935, 61.634, 19.019, 5.516, 1.892, 0.772, 0.404, 0.180, 0.05, 0.006].

Experimentally, the last 4 components of ***S*** (which have very small expectation values) will probably be realized as zeroes. A possible observed vector from this population at a sampling rate of 60% is [218, 62, 19, 6, 2, 1, 0, 0, 0, 0]. If equation 4 is applied to this observed vector, the estimate of ***A*** is given as [256.167, 103, 27.201, 46.913, -26.217, 21.611, 0, 0, 0, 0]. The one observed cluster of size 6 cannot, by the inversion of the upper triangular matrix **P**, be expressed as coming from a cluster greater than size 6. Instead, the inversion implies that the 1 observed cluster of size 6 came from 21 clusters of size 6 in the population. This implicit large number of clusters of size 6 would be expected to manifest as observed clusters of smaller sizes, but as they do not appear in the actual data, the estimator for the full vector ***A*** requires negative components to be consistent with equation 4.

In estimating the proportion of cases due to recent transmission, the estimates of interest are *A*_1_ and *M*, the number of singletons and the number of clusters, respectively. Even though the vector $\hat{A}$ is not a good estimator for ***A***, the first element of this vector and the sum of the elements provide good estimators for *A*_1_ and *M*. This is shown in Figures 1-3 above. The underlying theoretical reason for this observed stability is not investigated here.
