# Supplementary material for: Towards Eliminating Bias in Cluster Analysis of TB Genotyped Data
Source: PLoS One. 2012 Mar 29;7(3):e34109. doi: 10.1371/journal.pone.0034109 (PMC3315507; doi:10.1371/journal.pone.0034109)
Supplement: Supporting Information S3 — This file provides the algorithm for creating hypothetical populations for a given sample and code to reproduce the results in Table 1. (DOC) [file pone.0034109.s003.doc]

# Supporting Information S3

The hypothetical population data underlying the analyses were generated in an iterative scheme which optimizes the correspondence between the mean (simulated) observed cluster size distribution and the actual cluster size distribution in the data set for that city. A visual basic program was written that has as inputs, {*S*(*k*)} for (1 ≤ *k* ≤ *M*), and the sampling rate *r*. It has, for output, the hypothetical population cluster distribution {*A*(*k*)} for (1 ≤ *k* ≤ *M*).

The following algorithm produces the hypothetical population cluster distribution:

1. Set up an initial {*A*(k)} : Starting with {*S*(*k*)} let *A*(*k*) = *S*(*k*)/*r* where
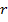
 is the sampling rate. This applies to all the *k* for which *S*(*k*) are known and 1 ≤ *k* ≤ *M*.
2. Sample the population comprising all cases represented by {*A*(k)} *s* times and take means to get {*R*(*k*)} and also calculate the standard deviations {*δ*(*k*)}. *s* can be a user specified parameter, typically 40 or more.
3. Compare *R*(*k*) with *S*(*k*) and set new values for the *A*(*k*): New values for the *A*(*k*) are produced by scaling *A*(*k*) i.e. *A*(*k*)*( *S*(*k*)/ *R*(k)). Do this for *k* = 1, 2, …, *M*.
4. Repeat steps 2 and 3 several times: Steps 2 and 3 are to be repeated a sufficient number of times i.e. until the differences *S*(*k*) – *R*(k) each have absolute values less than one standard deviation.

Table S1 below shows the particular cluster size distributions that were generated.

Table S1 Hypothetical population data

| **Cluster size** | **Cape Town** | **Alabama** | **San Francisco** | **Zaragoza** |
| --- | --- | --- | --- | --- |
| 1 | 644 | 1298 | 337 | 227 |
| 2 | 110 | 56 | 18 | 25 |
| 3 | 12 | 21 | 19 | 6 |
| 4 | 94 | 21 | 4 | 5 |
| 5 | 3 | 7 | 4 | 4 |
| 6 | 11 | 0 | 0 | 0 |
| 7 | 5 | 6 | 0 | 3 |
| 8 | 3 | 4 | 3 | 3 |
| 9 | 4 | 9 | 0 | 0 |
| 12 | 0 | 0 | 1 | 0 |
| 13 | 0 | 1 | 0 | 0 |
| 14 | 4 | 2 | 0 | 1 |
| 16 | 1 | 0 | 0 | 0 |
| 17 | 2 | 2 | 0 | 0 |
| 18 | 2 | 0 | 1 | 0 |
| 19 | 0 | 1 | 0 | 0 |
| 20 | 0 | 1 | 0 | 0 |
| 21 | 2 | 0 | 0 | 0 |
| 26 | 0 | 1 | 0 | 0 |
| 28 | 0 | 0 | 1 | 0 |
| 33 | 0 | 1 | 0 | 0 |
| 37 | 0 | 0 | 1 | 0 |
| 47 | 0 | 1 | 0 | 0 |
| 50 | 1 | 0 | 0 | 0 |
| 51 | 0 | 1 | 0 | 0 |
| 59 | 1 | 0 | 0 | 0 |
| 63 | 1 | 0 | 0 | 0 |
| 91 | 1 | 0 | 0 | 0 |
| 92 | 0 | 0 | 0 | 1 |
| 181 | 0 | 1 | 0 | 0 |
| 194 | 1 | 0 | 0 | 0 |

All analyses were done in R ([**www.r-project.org**](http://www.r-project.org/)**)**.

The R function that reproduces the results in Table 1:

estimateclusters <-

function( S, r , tol=1e-6, minrcond = 1e-6, alpha=0.95, trunc=Ntilde) {

## Sort out what size of P matrix we will use

Nobs <- (1:length(S))%*%S

N <- floor(Nobs/r)

Ntilde <- max( which( S != 0 ) )

S <- S[1:Ntilde]

S[ is.na( S ) ] <- 0

## Matrix Pinv

P <- matrix( 0, nrow=Ntilde, ncol=Ntilde )

for( i in 1:Ntilde ) {

P[i,i:Ntilde] <- dhyper( i, i:Ntilde, N - (i:Ntilde), Nobs )

}

Ntilde <- 1

while( (P[1,Ntilde] > tol) && (rcond( P[1:Ntilde,1:Ntilde,drop=FALSE] ) > minrcond) && (Ntilde < nrow( P ) ) )

Ntilde <- Ntilde + 1

Pfull <- P[1:Ntilde,1:Ntilde]

P <- Pfull[1:trunc,1:trunc]

Pinv <- solve(P) ## OK because of our truncation

Mmore <- sum( S[ -(1:trunc ) ] )

Stilde <- S[1:trunc]

## Estimate the number of clusters and the covariances

Ahat <- Pinv%*%Stilde

cov <- -Stilde %*% t( Stilde ) + diag( Stilde )

for( i in 1:trunc ) {

for( j in i:trunc ) {

for( m in i:trunc ) {

vec <- dhyper( j, j:trunc, N - m - (j:trunc ), Nobs - i )

cov[ i, j ] <- ( cov[ i, j ] + Ahat[ m ]*P[i,m] *

Ahat[j:trunc] %*% vec )

}

vec <- dhyper( j, j:trunc, N-2*(j:trunc), Nobs - i )

cov[ i, j ] <- cov[ i, j ] - Ahat[ j:trunc ] %*% ( P[i, j:trunc] * vec ) }

}

for( i in 2:trunc )

for( j in 1:(i-1) )

cov[ i, j ] <- cov[ j, i ]

## The estimators and their covariances

Mhat <- sum( Ahat ) + Mmore

A1hat <- Ahat[1]

covest <- Pinv%*% cov %*% t( Pinv )

Msd <- sqrt( sum( covest ) )

A1sd <- sqrt( covest[1,1] )

## Proportion of recent infections

pnhat = (N - A1hat)/N

pnminus1hat <- ( N - Mhat )/N

pnsd = A1sd/N

pnminus1sd = Msd/N

## Confidence intervals

MCI <- c( Mhat - qnorm( 1 - (1-alpha)/2 )*Msd, Mhat + qnorm( 1 - (1-alpha)/2 )*Msd )

A1CI <- c( A1hat - qnorm( 1 - (1-alpha)/2 )*A1sd, A1hat + qnorm( 1 - (1-alpha)/2 )*A1sd )

pnCI = c( pnhat - qnorm( 1 - (1-alpha)/2 )*pnsd, pnhat + qnorm( 1 - (1-alpha)/2 )*pnsd )

pnminus1CI =c( pnminus1hat - qnorm( 1 - (1-alpha)/2 )*pnminus1sd, pnminus1hat + qnorm( 1 - (1-alpha)/2 )*pnminus1sd )

pn1naive<-(Nobs-sum(S))/Nobs

pnnaive<-(Nobs-S[1])/Nobs

## Return

return(list(Mhat = Mhat, MCI = MCI, Msd = Msd, A1hat = A1hat, A1CI = A1CI, A1sd=A1sd, pnhat=pnhat, pnCI=pnCI, pnsd=pnsd, pnminus1hat = pnminus1hat, pnminus1CI = pnminus1CI, pnminus1sd = pnminus1sd,pnnaive=pnnaive,pn1naive=pn1naive, Ntilde=Ntilde))

}

The arguments initially needed in this function are
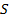
 and
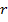
.

*S* is the sample vector of cluster frequencies (a histogram of observed cluster sizes). For example, the vector (100, 50, 20, 10, 5, 2, 2, 0, 1, 0, 0, 0, 1, 0, 1) represents a sample in which there were 100 singletons, 50 doublets etc.

The value
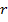
 is the percentage of TB diagnoses that were successfully typed, for example 70%.

The command to produce all the values in Table 1, for the vector and sampling rate above will be:

estimateclusters(S=c(100, 50, 20, 10, 5, 2, 2, 0, 1, 0, 0, 0, 1, 0, 1), r=0.7)

The output from this command is:

$Mhat

[1] 226.8779

$MCI

[1] 213.2553 240.5005

$Msd

[1] 6.950422

$A1hat

[1] 93.97432

$A1CI

[1] 66.29841 121.65022

$A1sd

[1] 14.12062

$pnhat

[,1]

[1,] 0.8303713

$pnCI

[1] 0.7804148 0.8803278

$pnsd

[,1]

[1,] 0.02548848

$pnminus1hat

[,1]

[1,] 0.5904731

$pnminus1CI

[1] 0.5658836 0.6150626

$pnminus1sd

[,1]

[1,] 0.01254589

$pnnaive

[,1]

[1,] 0.742268

$pn1naive

[,1]

[1,] 0.5051546

$Ntilde

[1] 15

The following R function generates a table that gives estimates for *M* and *A*_1_ for all the possible truncation cluster sizes. Note that the maximum value for “trunc” in this table is $\tilde{N}$, the maximum truncation cluster size for which the **P** matrix is still invertible.

make.table <-

function (S, r)

{

Ntilde <- estimateclusters(S, r )$Ntilde

Pinv <- estimateclusters(S, r )$Pinv

Mhat <- rep(0,(Ntilde-1))

Msd <- rep(0,(Ntilde-1))

A1hat <- rep(0,(Ntilde-1))

A1sd <- rep(0,(Ntilde-1))

A1n <- rep(0,(Ntilde-1))

Mn <- rep(0,(Ntilde-1))

for ( i in 2:Ntilde) {

a<-estimateclusters(S, r , trunc=i )

Mhat[i-1] <- a$Mhat

Msd[i-1] <- a$Msd

A1hat[i-1] <- a$A1hat

A1sd[i-1] <- a$A1sd

A1n[i-1]<-S[1]

Mn[i-1]<-sum(S)

}

list(table=data.frame(trunc=c(2:Ntilde),Mhat=Mhat, Msd=Msd,A1hat=A1hat ,A1sd=A1sd))

}

For the hypothetical sample mentioned above, the command and output will be:

make.table(S=c(100, 50, 20, 10, 5, 2, 2, 0, 1, 0, 0, 0, 1, 0, 1), r=0.7)

$table

trunc Mhat Msd A1hat A1sd

1 2 225.5524 6.967019 81.53787 13.63950

2 3 227.1595 6.932322 97.43635 13.94017

3 4 226.8068 6.957592 92.81133 14.10946

4 5 226.8849 6.949753 94.08351 14.11735

5 6 226.8709 6.951681 93.81247 14.11909

6 7 226.8772 6.950596 93.95402 14.12024

7 8 226.8772 6.950596 93.95402 14.12024

8 9 226.8778 6.950437 93.97273 14.12055

9 10 226.8778 6.950437 93.97273 14.12055

10 11 226.8778 6.950437 93.97273 14.12055

11 12 226.8778 6.950437 93.97273 14.12055

12 13 226.8779 6.950425 93.97399 14.12060

13 14 226.8779 6.950425 93.97399 14.12060

14 15 226.8779 6.950422 93.97432 14.12062

In this example, variability in the estimates is stable for different cut-offs. To choose any cut-off that is less than the default of $\tilde{N}$, run the first function above with, for example, trunc=7.

estimateclusters(S=c(100, 50, 20, 10, 5, 2, 2, 0, 1, 0, 0, 0, 1, 0, 1), r=0.7, trunc=7)
